# The relationship between sphingosine-1-phosphate receptor 2 and epidermal growth factor in migration and invasion of oral squamous cell carcinoma

**DOI:** 10.1186/s12935-023-02906-w

**Published:** 2023-04-10

**Authors:** Adjabhak Wongviriya, Richard M. Shelton, Paul R. Cooper, Michael R. Milward, Gabriel Landini

**Affiliations:** 1 School of Dentistry, Institute of Clinical Sciences, College of Medical and Dental Sciences, University of Birmingham, Edgbaston, Birmingham, UK; 2 Department of Oral Sciences, Sir John Walsh Research Institute, Faculty of Dentistry, University of Otago, PO Box 56, Dunedin, 9054 New Zealand

**Keywords:** S1PR2, EGF, Migration, Invasion, Crosstalk

## Abstract

Sphingosine-1-phosphate (S1P) is a lipid mediator and its binding to the S1P receptor 2 (S1PR2) is reported to regulate cytoskeletal organization. Epidermal growth factor (EGF) has been shown to induce migration and invasion in tumour cells. Since binding of S1P to S1PR2 and EGF to the EGF receptors exhibit some overlapping functionality, this study aimed to determine whether S1PR2 was involved in EGF-induced migration and invasion of oral squamous cell carcinoma (OSCC) lines and to identify any potential crosstalk between the two pathways. Migration was investigated using the scratch wound assay while invasion was studied using the transwell invasion and multicellular tumour spheroid (MCTS) assays. Activity of Rac1, a RhoGTPase, was measured using G-LISA (small GTPase activation assays) while S1P production was indirectly measured via the expression of sphingosine kinase (Sphk). S1PR2 inhibition with 10 µM JTE013 reduced EGF-induced migration, invasion and Rac1 activity, however, stimulation of S1PR2 with 10 µM CYM5478 did not enhance the effect of EGF on migration, invasion or Rac1 activity. The data demonstrated a crosstalk between EGF/EGFR and S1P/S1PR2 pathways at the metabolic level. S1PR2 was not involved in EGF production, but EGF promoted S1P production through the upregulation of Sphk1. In conclusion, OSCC lines could not migrate and invade without S1PR2 regulation, even with EGF stimulation. EGF also activated S1PR2 by stimulating S1P production via Sphk1. The potential for S1PR2 to control cellular motility may lead to promising treatments for OSCC patients and potentially prevent or reduce metastasis.

**Supplementary Information:**

The online version contains supplementary material available at 10.1186/s12935-023-02906-w.

## Introduction

Sphingosine-1-phosphate (S1P) is a bioactive lipid that regulates various cellular activities of the immune, cardiovascular and nervous systems [1, 2]. In cancer, S1P has been reported to regulate proliferation, differentiation, migration, invasion, angiogenesis and cell survival [3–8]. S1P is derived from the catabolism of intracellular ceramide by ceramidase in the cytosol which converts it into sphingosine. This is then converted into S1P by sphingosine kinase (Sphk) which is exported extracellularly where it can bind to S1P receptors (S1PR1-5) to mediate various activities [1].

Thus far, studies have aimed to determine the function of the S1P receptor subtypes in several different cells. To date, only the function of S1PR1 has been established, and evidence for the function and role of the other receptor subtypes remains limited. S1PR2 has been reported to regulate cell growth, survival, angiogenesis and adhesion [1, 9], although it has been most studied in relation to cell mobilisation [5–7, 10, 11]. The effects of S1P/S1PR2 are cell type dependent; and it has been reported to induce tumour suppression in human anaplastic thyroid cancer [6], glioblastoma [12], B16 melanoma [11] and neuroblastoma cells [10] but also to accelerate metastasis of human adenocarcinoma [13] and oral squamous cell carcinoma (OSCC) cells [5]. In relation to invasion, S1PR2 was reported to activate Rac1 [12, 14, 15], a RhoGTPase that induced actin polymerization and lamellipodia extension during cell migration [16].

EGF is another cytokine that controls a variety of cellular activities [17–20]. In OSCC, EGF binding to its receptors was reported to promote proliferation, migration, invasion [17, 18] and epithelial-mesenchymal transition [19]. There is evidence that EGF treatment can drive cell migration through the stimulation of Rac1 [21] and also there is a functional overlap between S1P/S1PR2 and EGF/EGF receptors pathways in controlling cellular motility [22–25]. Nevertheless, no studies have determined the effect of S1PR2 on EGF-induced invasion previously nor determined the crosstalk between S1P/S1PR2 and EGF/ EGF receptors pathways.

New therapies targeting specific molecules related to individual neoplastic properties might be able to fill gaps in current approaches for the management of cases, where conventional treatments have been exhausted. Since the major cause of cancer deaths is distant metastasis, new approaches that prevent invasion have the potential to improve prognosis and outcome.

Additionally, although conventional treatment of OSCC includes excision, chemo- and radio-therapy some cases of recurrence may not be suitable for further conventional treatment. In such situations, a targeted medicine specific to molecules controlling the neoplastic behaviour may improve treatment outcomes. Since the major cause of cancer death is distant metastasis, a medicine that prevents invasion potentially improves the prognosis. For this reason, the present study aimed to investigate the involvement of S1PR2 in EGF-induced migration and invasion in three OSCC cell lines as well as to identify any potential crosstalk between S1P/S1PR2 and EGF/EGF receptor signalling.

## Materials and methods

Human recombinant EGF (Gibco, UK) was prepared to a final concentration of 20 ng/ml in cell culture media as this concentration has been reported to be optimal for activating squamous carcinoma cells [26].

JTE013, [1-[1,3-dimethyl-4-(2-methylethyl)-1 H-pyrazolo[3,4-b]pyridin-6-yl]-4-(2,6-dichloro-4-pyridinyl)-semicarbazide] (Tocris bioscience, UK) is a S1PR2 antagonist while CYM5478, [1-[2-[2,5-dimethyl-1-(phenylmethyl)-1 H-pyrrol-3-yl]-2-oxoethyl]-5-(trifluoromethyl)-2(1 H)-pyridinone] (Cayman Chemical, UK), is a S1PR2 agonist. For the experimental work, both JTE013 and CYM5478 were dissolved in dimethyl sulfoxide (DMSO) (Sigma, UK) and freshly diluted in media to 10 µM immediately prior to use.

### Cell cultures

Three human OSCC lines, H357 (ECACC 06092004, from tongue), H400 (ECACC 06092006, from the alveolar process) and H413 (ECACC 06092007, from buccal mucosa) [17], passage numbers 35–45, were cultured in Dulbecco′s modified Eagle′s medium/Ham′s nutrient mixture F12, 1:1 mixture (DMEM) (SAFC Biosciences, UK) supplemented with 0.25 µg/ml hydrocortisone (Sigma, UK), 2 mM L-glutamine (Sigma, UK), 98 units/ml penicillin-streptomycin solution containing 98 µg/ml streptomycin (Sigma, UK) and 10% foetal bovine serum (FBS) (Biosera, UK). Cultures were maintained in a HERAcell 150i incubator (Thermo Scientific, UK) at 37^°^C in an atmosphere of 5% CO_2_. Media was replenished every second day until cultures were approximately 70% confluent.

### Scratch wound migration assay

OSCC lines were cultured in 6-well plates until confluent. Cultures were treated with 8 µg/ml mitomycin C (Merck, UK) for 2 h to inhibit proliferation. Scratch wounds were generated using sterile disposable plastic pipette tips (10 mm diameter) and washed with phosphate buffered saline (PBS) (Sigma, UK) before being replenished with FBS-free media containing 20 ng/ml EGF, 20 ng/ml EGF with 10 µM JTE013 and 20 ng/ml EGF with 10 µM CYM5478. Four images were captured with a digital camera (Nikon D5100, Japan) (resolution: 1 pixel = 0.58 μm) immediately and at 24 h after generating scratch wounds, using phase contrast microscopy (Nikon Eclipse TE300, Japan). Images were analysed by measuring the gap area of the wounds using the MRI wound healing tool plugin for ImageJ software [27]. Experiments were performed with three biological replicates.

### Transwell invasion assay

Thirty microlitres of 0.5 mg/ml rat tail collagen type I (Cultrex, UK) were added to a 24-well transwell insert (Greiner Bio-One, UK) and solidified at 37 °C for 30 min. Three hundred microlitres of FBS-free media containing 2.5 × 10^4^ cells were added to the tissue culture inserts, while 500 µl of media containing 20% FBS was added to the lower compartment and cultures maintained at 37 °C in 5% CO_2_ for 48 h. The collagen and cells on the upper surface of the membrane were removed using a cotton swab. Cells on the lower side of the membrane were stained with calcein AM (Invitrogen, UK) and visualised using fluorescence microscopy (Nikon Eclipse TE300, Japan). Ten images were captured with digital camera (Nikon, Japan) (resolution: 1 pixel = 0.24 μm)   and cell counts were performed with experiments performed in triplicate.

### Multicellular tumour spheroids

Tumour spheroids were generated using the hanging drop method for 24 h (approximately 500 cells/spheroid) with each spheroid embedded in 3 mg/ml rat tail collagen type I (Cultrex, UK). Samples were treated with 8 µg/ml mitomycin C for 2 h to inhibit proliferation, then washed three times with PBS before replenishing with media according to the experimental groups including FBS-free media, media containing EGF concentrations, media containing EGF with 10 µM JTE013 and media containing EGF with 10 µM CYM5478 [26]. The tumour spheroids were incubated with calcein AM (Biotium, UK) for 30 min without exposure to light. Culture images were captured using confocal microscopy (Zeiss, Germany) (resolution: 1 pixel = 1.53 μm) on the same day spheroids were embedded as well as on experimental day 2. Experiments were undertaken with three biological replicates with ten spheroids for each experiment. ImageJ software was used to determine invasion parameters: number of cell clusters, maximum invading distance and circularity of the main cluster. The circularity of the main cluster was computed using the formula: Circularity = 4πArea/Perimeter^2^ (Area refers to area of the main cluster of the spheroid; Perimeter refers to the length of the cluster boundary) and the maximum invading distance referred to the largest distance between any two points in a set [28].

### Enzyme-linked immunosorbent assay

OSCC lines were treated with either the S1PR2 antagonist or agonist for 48 h. The levels of EGF and TGF-β1 were measured in the culture supernatants using ELISA (Quantikine ELISA Kit, R&D Systems, USA) following the manufacturer’s instructions. Three biological replicates were performed.

### Semiquantitative PCR

To compare the expression profile of S1PR1-5, three OSCC lines were cultured in T25 flasks until confluent. Total RNA was extracted using the RNeasy Mini Kit (Qiagen, UK). Single stranded cDNA was synthesized from 2 µg of RNA using the Tetro kit (Bioline, UK). The amplification process was performed using the following conditions: denaturation at 94°C for 5 min; annealing (25 cycles) at 94°C for 20 sec, 60°C for 20 sec and 72°C for 20 sec; and extension at 72°C for 10 min, respectively [29]. The amplification primers sequences used were: S1PR1 F, 5’-GCCCAGTGGTTTCTGCGGGAA-3’, S1PR1 R, 5’-ACCAAGGAGTAGATCCTGCAGTA-3’; S1PR2 F, 5’-ACCAAGGAGTAGATCCTGCAGTA-3’, S1PR2 R, 5’-GCAACAGAGGATGACGATGA-3’; S1PR3 F, 5’-CTCAGGGAGGGCAGTATGT TC-3’, S1PR3 R, 5’-GGACTTGACCAGGAAGTAGATGCG-3’; S1PR4 F, 5’-TCCAGCCTTCTG CCCCTCTAC-3’, S1PR4 R, 5’- CAGGGCCAGGATCCAGTCCAT-3’; and S1PR5 F, 5’-GCCGGTGAGCGAGGTCATCGT-3’, S1PR5, R, 5’- TAGGCCTTGGCGTAGAGCGG − 3’. The PCR products were analysed by gel electrophoresis in 1.5% agarose gel and visualised using the G:BOX Syngene image analyser (Syngene, UK). Relative RNA levels between samples were determined from gel quantification by normalising to the level of GAPDH, the housekeeping gene. Experiments were performed with three biological replicates.

### Quantitative real time PCR

Cultures were incubated with media containing either 20 ng/ml EGF; or 20 ng/ml EGF plus 10 µM JTE013 or 10 µM CYM5478 for 48 h. Total RNA was extracted using the RNeasy Mini Kit (Qiagen, UK). Single stranded cDNA was synthesized from 2 µg of RNA using the Tetro kit (Bioline, UK). cDNA was used as a template for quantitative real-time polymerase chain reaction (qRT-PCR) using a LightCycler 480 SYBR Green I Master kit (Roche Diagnostics, UK). The amplifying process included 5 min of initial denaturation at 95°C followed by 45 cycles of amplification (each cycle consisted of denaturation at 95°C for 10 sec, annealing at 60°C for 15 sec, and elongation at 72°C for 10 sec). The relative changes of RNA levels of Sphk1 and Sphk2 were normalised to the level of the best housekeeping gene identified for each cell line (YWAHZ for H357, GAPDH for H400 and B2M for H413). Primers sequences used were: Sphk1 F, 5’- GCTGCGAAGTTGAGCGAAAA-3’, Sphk1 R, 5’- CCCGCTGGATCCATAACCTC-3’; and Sphk2 F, 5’-CTAGTCGGGGCATCTGGAAA-3’, Sphk2 R, 5’- CTCACTGTCCTGGCCTGAC-3’. Experiments were undertaken with three biological replicates.

### G-LISA activation assay

Rac1 activity was measured using the G-LISA activation assay (Cytoskeleton, UK). OSCC lines were cultured in T25 flasks until reaching 50% confluence. Cultures were serum starved with 0.5% FBS media for 1 day and FBS-free media for 1 more day. S1PR2 was pre-treated with 10 µM JTE013 or 10 µM CYM5478 for 15 min. Cultures were incubated with 20 ng/ml EGF for 2 min. Cultures were lysed and the total protein was diluted to a concentration of 0.3 mg/ml. The G-LISA was performed according to the manufacturer’s protocol with luminescence measured using a Spark® multimode microplate reader (Tecan, Switzerland).

### Statistical analysis

Differences between groups were statistically analysed using ANOVA followed by post-hoc Tukey tests using GraphPad Prism (Version 9.0.0). P-values of less than 0.05 were considered statistically significant.

## Results

### The expression of S1PRs

**Fig. 1:**
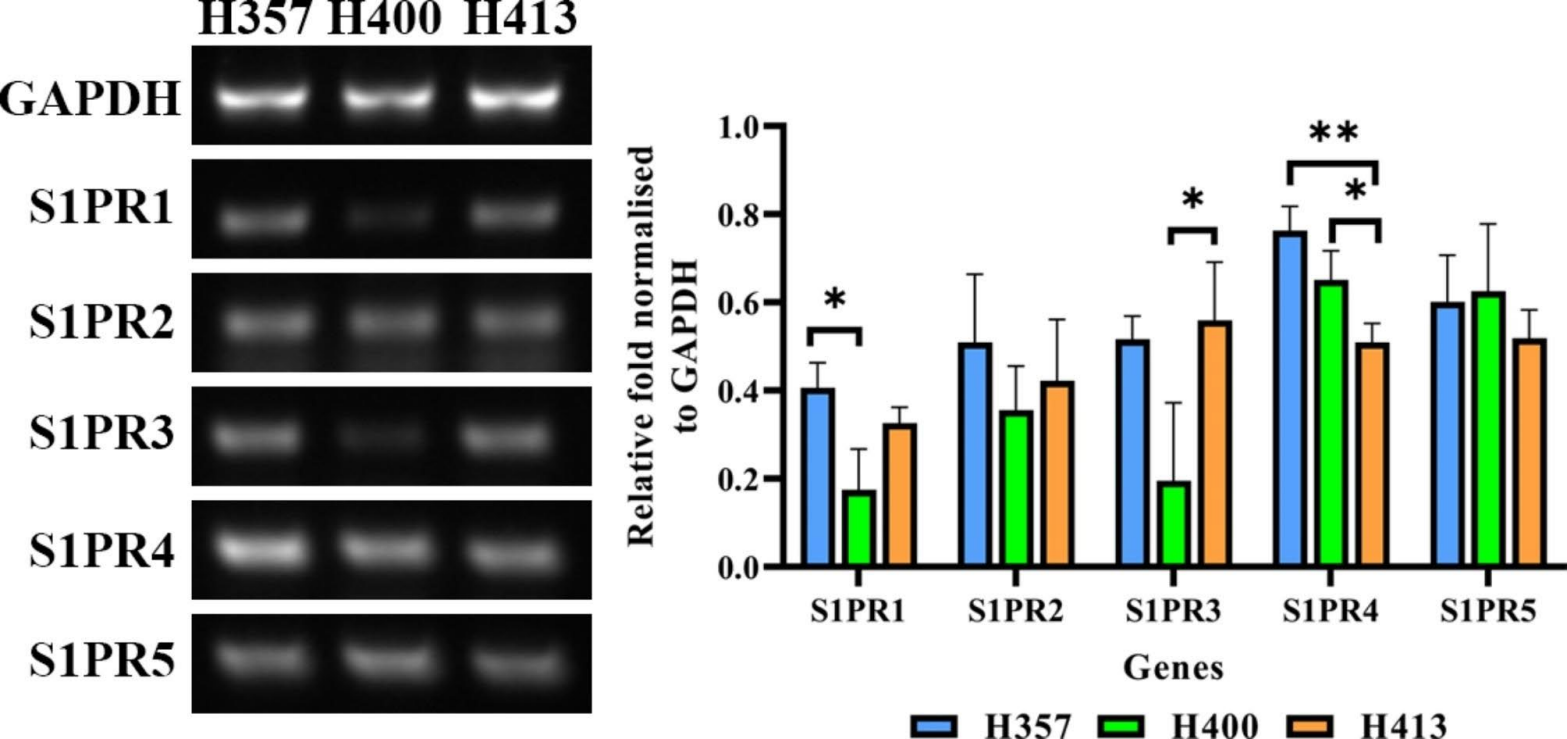
The S1PR profile in H357, H400 and H413 cells, determined using semiquantitative PCR. Three OSCC lines expressed five S1PR subtypes. The expression of S1PR1 of H400 was lower than in H357, while its S1PR3 expression was lower than in H413 cells. The expression of S1PR4 of H413 was lower than H357 and H400 cells. There was no difference in S1PR2 and S1PR5 expression between three cell lines. (N = 3; ANOVA followed by post-hoc Tukey tests, * = p-value < 0.05, ** = p-value < 0.01, data presented as mean ± 1 SD.)

The three OSCC lines expressed S1PR1-5 (Fig. 1). The expression of S1PR1 in H357 was higher than that of H400 (p < 0.05, ANOVA). No statistical differences in S1PR2 and S1PR5 expression levels between the three OSCC lines were identified. H413 cells expressed higher S1PR3 levels than that of H400 cells (p < 0.01, ANOVA), but expressed lower S1PR4 levels than H357 cells (p < 0.01, ANOVA) and H400 (p < 0.05, ANOVA).

### Effects of S1PR2 on EGF-induced migration

**Fig. 2:**
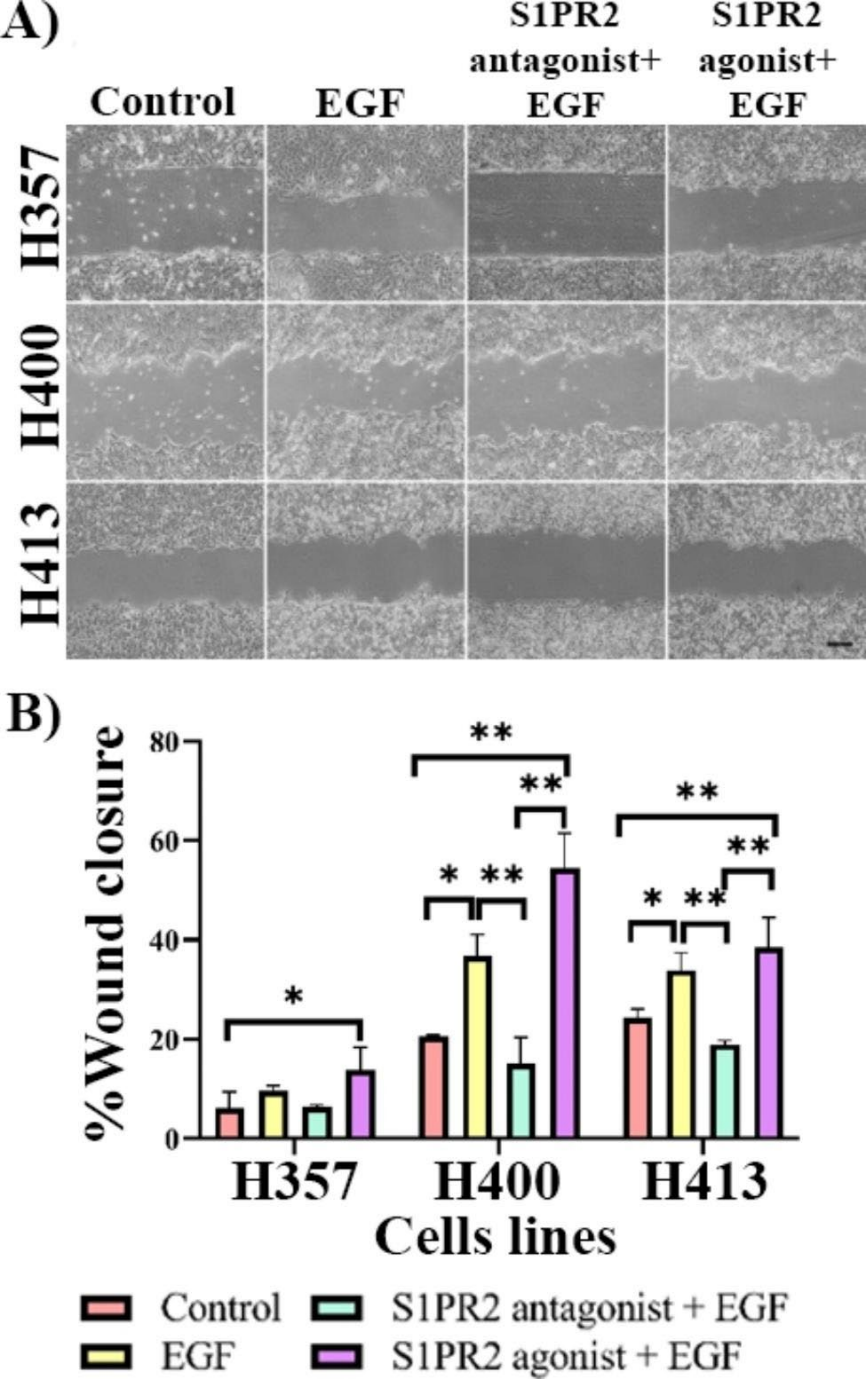
The effect of S1PR2 on EGF-induced migration of OSCC cells was determined using the scratch wound assay. **(A)** Representative images captured using phase-contrast microscopy. **(B)** Scratch wound cultures were performed for 24 h, and EGF stimulated migration of H400 and H413 cell lines. This effect of inducing migration significantly decreased when S1PR2 was inhibited, but this did not increase when cells received both the EGF and S1PR2 agonist. H357 cells showed a similar trend to the other two cell lines, however a statistically significant difference in percentage gap closure was only identified between the control and group treated with both EGF and the S1PR2 agonist. (Scale bar shown represents 200 μm; Three biological replicates were performed with N = 4 for each experiment; ANOVA followed by *post-hoc* Tukey test, * = p-value < 0.05 and ** = p-value < 0.01, data presented as mean ± 1 SD.)

The activation of S1PR2 can reportedly induce migration in a similar manner to EGF [5], therefore the effects of S1PR2 on EGF-induced migration in OSCC cells was examined. Data showed that 20 ng/ml EGF increased migration of H400 and H413 cells compared with controls (p < 0.05, ANOVA) (Fig. 2). When the S1PR2 antagonist was added to the EGF treatment, migration significantly reduced (p < 0.01, ANOVA) to the same level as that of the controls. However, migration did not increase when cells were treated with both EGF and the S1PR2 agonist and the H357 cell line showed (Fig. 2B) the slowest rate of migration. Although EGF and S1PR2 treatments caused changes in rate of wound closure which was similar in H400 and H413 cultures this was not statistically different except between the control group and the group treated with both EGF and the S1PR2 agonist. These data were consistent with the transwell migration assay which showed that 20 ng/ml EGF treatment increased migration. Inhibition of S1PR2 decreased this effect, however stimulation of S1PR2 did not enhance this effect (data not shown).

### Effect of S1PR2 on EGF-induced invasion

**Fig. 3:**
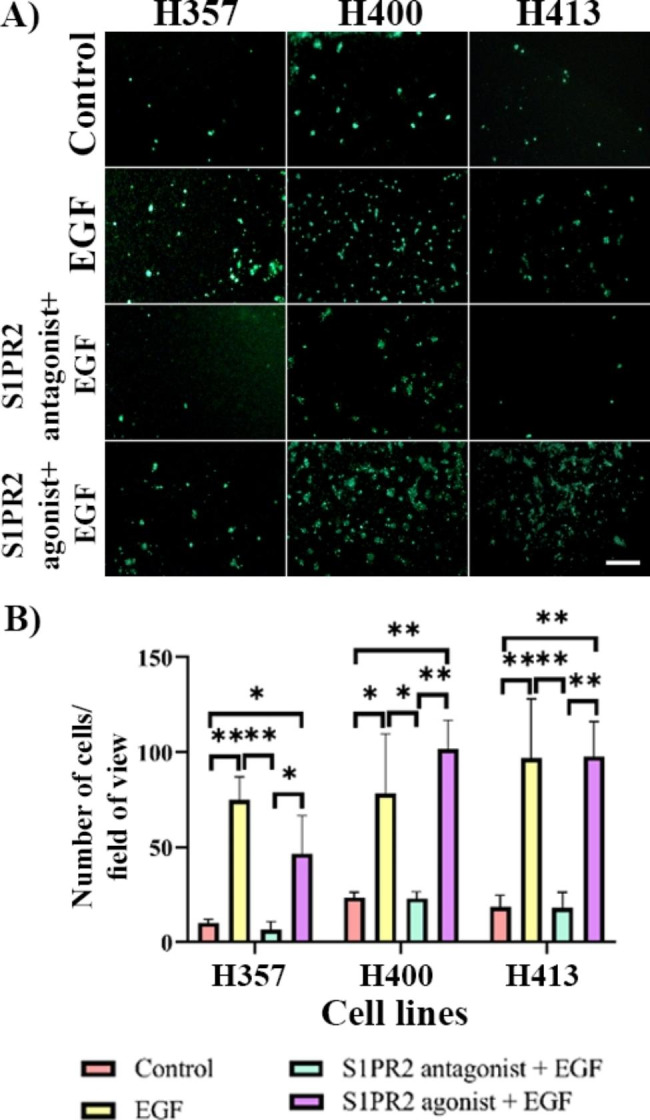
OSSC lines were incubated with EGF and S1PR2 treatments for 48 h. **(A)** Representative images of transwell invasion assay according to the different treatments, captured using fluorescent microscopy. **(B)** EGF increased invasion of all three cell lines. These effects decreased when S1PR2 was not activated. However, when S1PR2 was activated combined with EGF treatment, invasion did not increase. (Scale bar represents 200 μm. N = 30; ANOVA followed by post-hoc Tukey tests, * = p < 0.05, ** = p < 0.01.)

The effect of S1PR2 on EGF-induced invasion was investigated using a transwell invasion assay, which showed a similar trend to that previously identified (Fig. 3). Invasion was increased following EGF treatment 3–7 fold, dependent on the cell line (p < 0.05 in H357, p < 0.01 in H400 and H413 cultures, ANOVA). This effect was significantly decreased when S1PR2 was inhibited to the same level as the control group (p < 0.05 in H400, p < 0.01 in H357 and H413 cultures, ANOVA). When the S1PR2 agonist was combined with EGF treatment, no increase in the invasion of any of the three cell lines was identified.

**Fig. 4:**
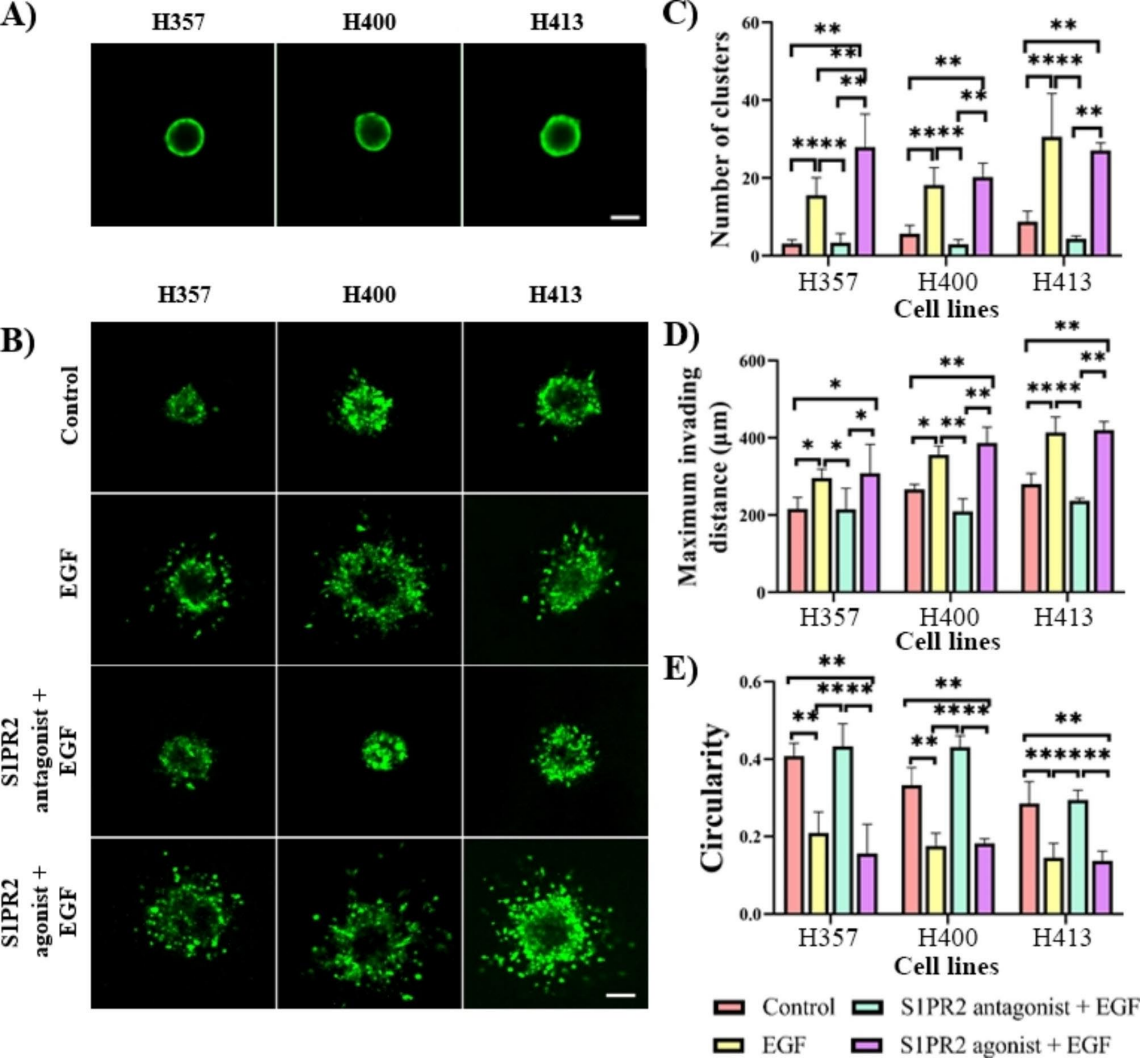
Multicellular tumour spheroids assay determining the effect of EGF and S1PR2 **(A)** MCTSs of H357, H400 and H413 cell lines after hanging drop culture for 1 day. Spheroids consisted of approximately 500 cells. The size and circularity of the spheroids on the embedding day were similar. **(B)** Images of spheroids embedded in 3 mg/ml collagen after incubation with treatments for 48 h. **C-E)** EGF at 20 ng/ml resulted in an increase in both spheroid fragmentation (note the number of clusters) and the distance of invasion as well as a decrease in circularity of the main cluster. The effects of EGF on these characteristics of invasion decreased following inhibition of S1PR2. These morphological parameters did not change after treatment following the addition of the S1PR2 agonist following EGF treatment. (Scale bar shown represented 100 μm; three biological replicates were performed with N = 10 for each experiment; ANOVA followed by post-hoc Tukey tests, * = p < 0.05, ** = p < 0.01, data presented as mean ± 1 SD.)

The transwell invasion assay data indicated invasion potential, however this may have only a limited correlation with the tissue invasion that occurs in vivo. Therefore, a MCTS model to assay invasion was also studied (Fig. 4). Three parameters were used to describe invasion characteristics: (a) the number of cell clusters represented by the fragmentation of the tumour at a given time, (b) the maximum invading distance of the culture (the largest distance between migrating cell clusters), and (c) the circularity of the main (largest) cluster as a representation of the shape of spheroid (where fractional values from 0 to 1 indicate an increasingly circular shape, although the ideal circularity of 1 cannot be obtained from shapes represented on discrete square lattices). Following incubation with the treatments, tumour spheroids were embedded in 3 mg/ml collagen for 48 h. EGF induced high spheroid fragmentation (4.9 fold in H357, 3.2 fold in H400 and 3.5 fold in H413 cultures, p < 0.01, ANOVA) and resulted in larger distances between the most distant invading cells compared with controls (1.4 fold in H357 and 1.3 fold in H400, p < 0.05 and 1.5 fold in H413, p < 0.01; ANOVA). When focusing on the main cluster, EGF treatment caused a reduction in the circularity of the spheroids from 0.40 to 0.20 in H357 cells; from 0.33 to 0.17 in H400 and from 0.28 to 0.14 in H413 cells (p < 0.01, ANOVA). When S1PR2 was inhibited, the effect of EGF resulted in a 5.3–7.8 fold reduction in the number of invading clusters (p < 0.01, ANOVA), 1.4–1.7 fold reduction of the maximum invading distance (p < 0.05 in H357, p < 0.01 in H400 and H413; ANOVA) and an increase in the circularity of the main cluster (p < 0.01, ANOVA) suggesting that the inhibition of S1PR2 reduced invasiveness. There was no statistical difference between the three parameters in the group treated only with EGF and the group treated with both the S1PR2 agonist and EGF.

### S1PR2 inhibition reduces EGF-induced Rac1 activity

**Fig. 5:**
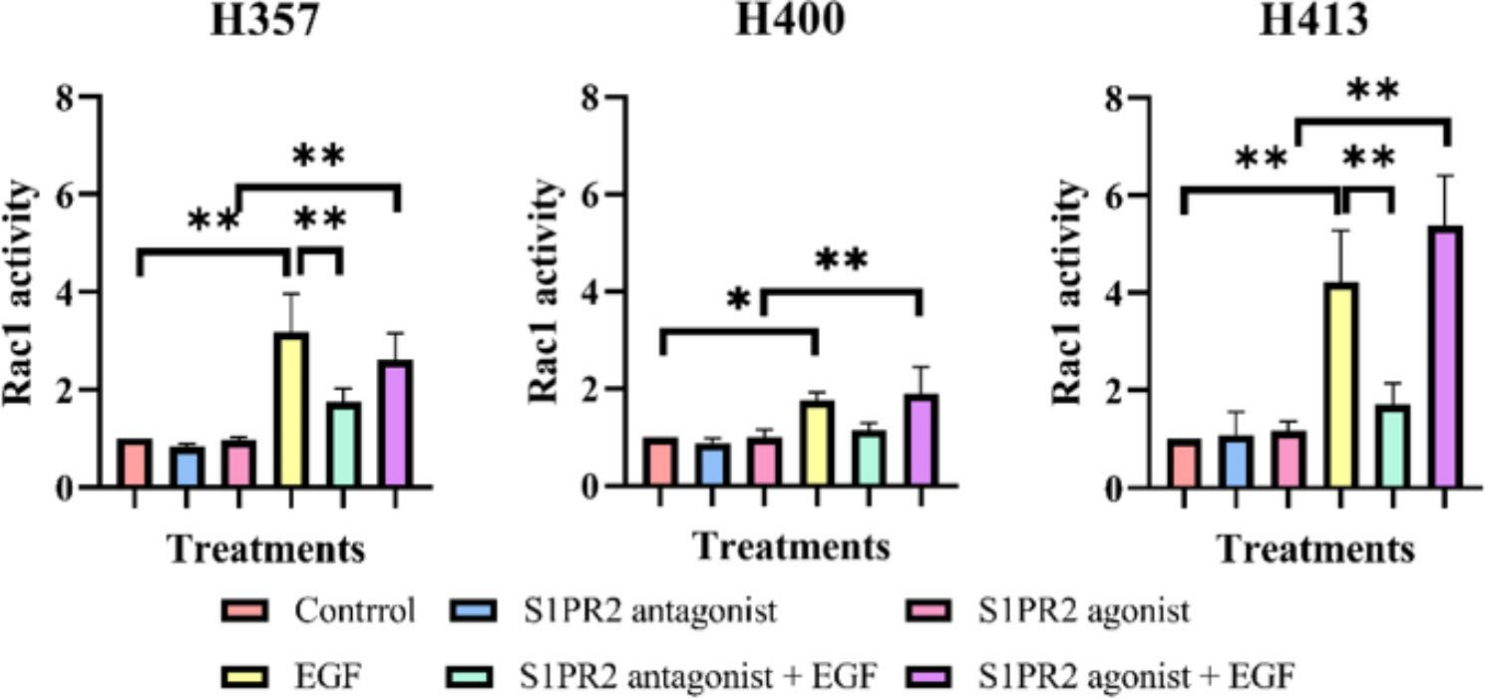
Rac1 activity determined by G-LISA. The three cell lines showed similar patterns of activity. The S1PR2 antagonist or agonist did not induce any statistically significant changes in Rac1 activity. EGF treatment alone increased Rac1 activity, but not when S1PR2 signaling was additionally suppressed. (N = 3; ANOVA followed by post-hoc Tukey tests, * = p-value < 0.05, ** = p-value < 0.01, data presented as mean ± 1 SD.)

After establishing the requirement for S1PR2 on EGF-induced migration and invasion, the involvement of the Rac1, a RhoGTPase implicated in cytoskeleton transduction, was investigated [17]. Neither blocking nor enhancing S1PR2 affected Rac1 activity (Fig. 5). After treatment of cells with EGF for 2 min, Rac1 activity rapidly increased by approximately 3.0 fold in H357 (p < 0.01, ANOVA), 1.7 fold in H400 (p < 0.05, ANOVA) and 4.0 fold in H413 (p < 0.01, ANOVA) cultures. However, EGF failed to stimulate Rac1 activity in the three lines when S1PR2 was suppressed. The group treated with both the S1PR2 agonist and EGF showed higher activity of Rac1 compared with the group treated with the S1PR2 agonist alone at approximately 2.7 fold in H357 (p < 0.01, ANOVA), 1.9 fold (p < 0.01, ANOVA) and 4.5 fold in H413 (p < 0.01, ANOVA) cultures, however, these increases were not statistically different to those of cultures treated with EGF alone. These data implied that S1PR2 was required for Rac1 activation, although the main molecule responsible for the increased activity was EGF.

### Crosstalk between S1P and EGF

The data described above indicated that S1PR2 was involved in the motility of H357, H400 and H413 cells. Consequently, the potential crosstalk between S1P/S1PR2 and EGF/EGF receptors signalling was investigated, and the effect of S1PR2 on the production of EGF and TGF-β1, which was reported to transactivate EGF receptors [30], was determined using ELISA. Notably, the baseline levels of EGF and TFG-β1 production in the three cell lines was at the picogram level and was not affected by either S1PR2 antagonist or agonist treatments for 48 h (data not shown).

**Fig. 6:**
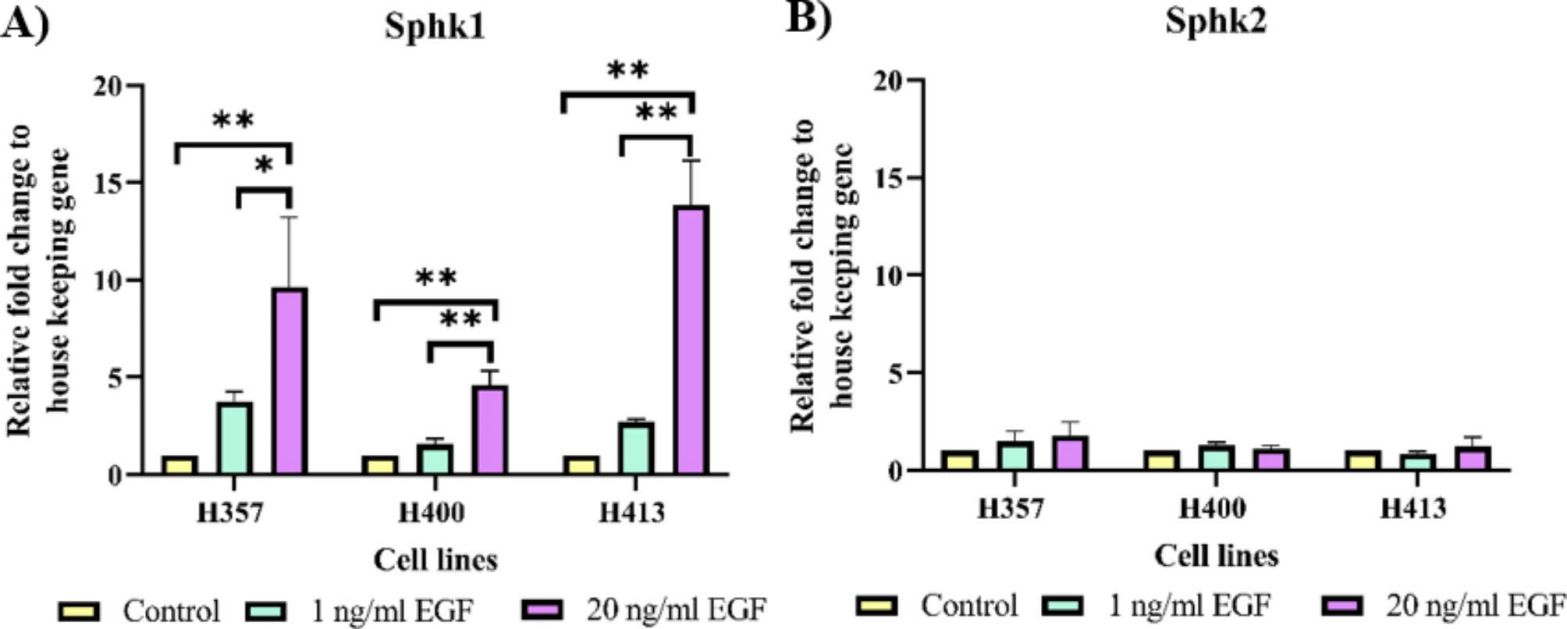
The three cell lines were treated with 1 ng/ml EGF and 20 ng/ml EGF for 48 h before performing real time RT-PCR analysis. Expression of Sphk1 and Sphk2 was calculated as relative fold change and normalised to housekeeping gene expression. **(A)** Sphk1 expression of three lines increased according to the EGF concentration. **(B)** Expression of Sphk2 did not change with treatment (N = 3; ANOVA followed by post-hoc Tukey tests, * = p-value < 0.05, ** = p-value < 0.01, data presented as mean ± 1 SD.)

The crosstalk between these receptors was also investigated by treating the cell lines with the two concentrations of EGF at 1 ng/ml and 20 ng/ml. S1P production was indirectly measured through gene expression analysis for the enzymes Sphk1 and Sphk2 which convert sphingosine into S1P. Sphk1 expression of the three cell lines increased according to the EGF concentration (Fig. 6A). EGF at 20 ng/ml induced significantly higher expression of Sphk1 than the control, approximately 9.6 fold in H357, 4.6 fold in H400 and 13.8 fold in H413 cells (p < 0.01, ANOVA). In contrast, the expression of Sphk2 in the three cell lines did not change significantly following the EGF treatments (Fig. 6B).

## Discussion

### Effects of S1PR2 on EGF-induced migration and invasion

Both EGF/EGF receptors and S1P/S1PR2 pathways are known to affect cell motility [5–7, 10, 11, 18, 26]. The present study demonstrated that S1PR2 affected EGF in controlling migration and invasion. Transwell invasion assays provided quantitative data but were limited in providing information about invasive features, such as changes in tumour morphology or mode of invasion. The MCTS assay could not distinguish whether the invasion may have been due to proliferation or migration, influencing whether the data from the transwell invasion assay was meaningful [26]. EGF was a strong inducer of migration and invasion for H357, H400 and H413 cells. S1PR2 activation also promoted invasion, fragmentation of the spheroids and caused the spheroid cluster to become morphologically irregular as a result of cells budding out before detaching from the spheroid (and subsequently spreading). The data also indicated that although EGF could increase migration and invasion, it required control by S1PR2. This finding was consistent with a previous study using transwell assay which demonstrated that breast cancer cells required regulation of S1PR2 for invasion [23]. That study also indicated that S1PR2 regulated cytoskeleton activity through the activation of ezrin, radixin and moesin (ERM), a family of proteins which enable linking of cortical actin to the plasma membrane, consequently cellular mobility was not achieved without signalling from this receptor. Interestingly, although the effect of EGF in driving migration and invasion was reduced when S1PR2 was inhibited, this effect was not increased following the addition of the S1PR2 agonist after cells had been treated with EGF.

### S1PR2 inhibition reduces EGF-induced Rac1 activity

Reorganisation of the cytoskeleton is key to cell motility. To examine how both EGF and S1PR2 may act to coordinate and regulate cell motility, downstream signalling of the RhoGTPase, Rac1, was further investigated. Activation of Rac1 was caused by EGF/EGF receptors stimulation rather than via the S1P/S1PR2 axis, however to stimulate Rac1 activity, a signal from S1PR2 was required. This was in agreement with a previous study, reporting the inability to detect Rac1 changes following S1P activation [31]. Notably, other studies report that S1PR2 inhibition could either activate Rac1 [32–34] or inactivate it [12, 14, 15]. Nevertheless, among those reports on Rac1 activity suppression following S1PR2 treatment, one study [12] showed that this mechanism did not involve S1P-mediated inhibition of migration, indicating that the activation of Rac1 following any treatment may be coincidental. In the OSCC lines studied here, Rac1 appeared to be inactivated after S1PR2 inhibition, however this was noted only when cells were incubated with the treatment for two minutes. A later study reported two waves of Rac1 activation following EGF treatment: the first wave occurred after treatment with EGF for two to five minutes and the second wave occurred after treatment for 6 to 12 h [35]. These two waves of Rac1 activation were reported to be due to EGF activating different guanine exchange factors (GEFs), proteins that activate the Rho GTPases: vav guanine nucleotide exchange factor 2 (VAV2) and Rho guanine nucleotide exchange factor 4 (Asef) for the first wave and TIAM Rac1 associated GEF 1 (TIAM1) for the second wave [35]. Therefore, to link the invasion features from the MCTS model, Rac1 activity monitoring should be performed.

### Crosstalk between S1P and EGF

Notably, EGF/EGF receptors and S1P/S1PR2 may neither have a separate cascade nor individually regulate Rac1 since the EGF effect on migration, invasion and Rac1 activity did not increase when both receptors were activated. Several studies have postulated mechanisms that explain the crosstalk of metabolism between S1P and EGF [22–24, 36, 37]. One such model proposed that Sphk expression could be rapidly increased by a variety of growth factors [25] including platelet-derived growth factor [38], vascular endothelial growth factor [39], EGF [36], tumour necrosis factor alpha [40] as well as some enzymes, such as acid ceramidase [41]. This mechanism may cause a translocation from the cytosol to the plasma membrane where Sphk becomes activated and converts sphingosine into S1P. Subsequently, S1P binds to S1PRs and triggers various cellular activities [25].

Another proposed model of breast cancer progression [22] involves a three-way-relationship between oestrogen, S1P and EGF. Oestrogen activates Sphk to produce S1P which is exported into the intercellular space and binds to S1PRs, mainly via S1PR3 and activates downstream signalling molecules, converting heparin-binding EGF-like growth factor (HB-EGF) into EGF. Consequently, EGF is exported into the intercellular space where it can bind to EGF receptors [22]. This mechanism has also been observed in a study using vascular smooth muscle cells, however EGF production was mainly induced through the activation of S1PR1 and possibly by S1PR3 and S1PR5 [37].

**Fig. 7:**
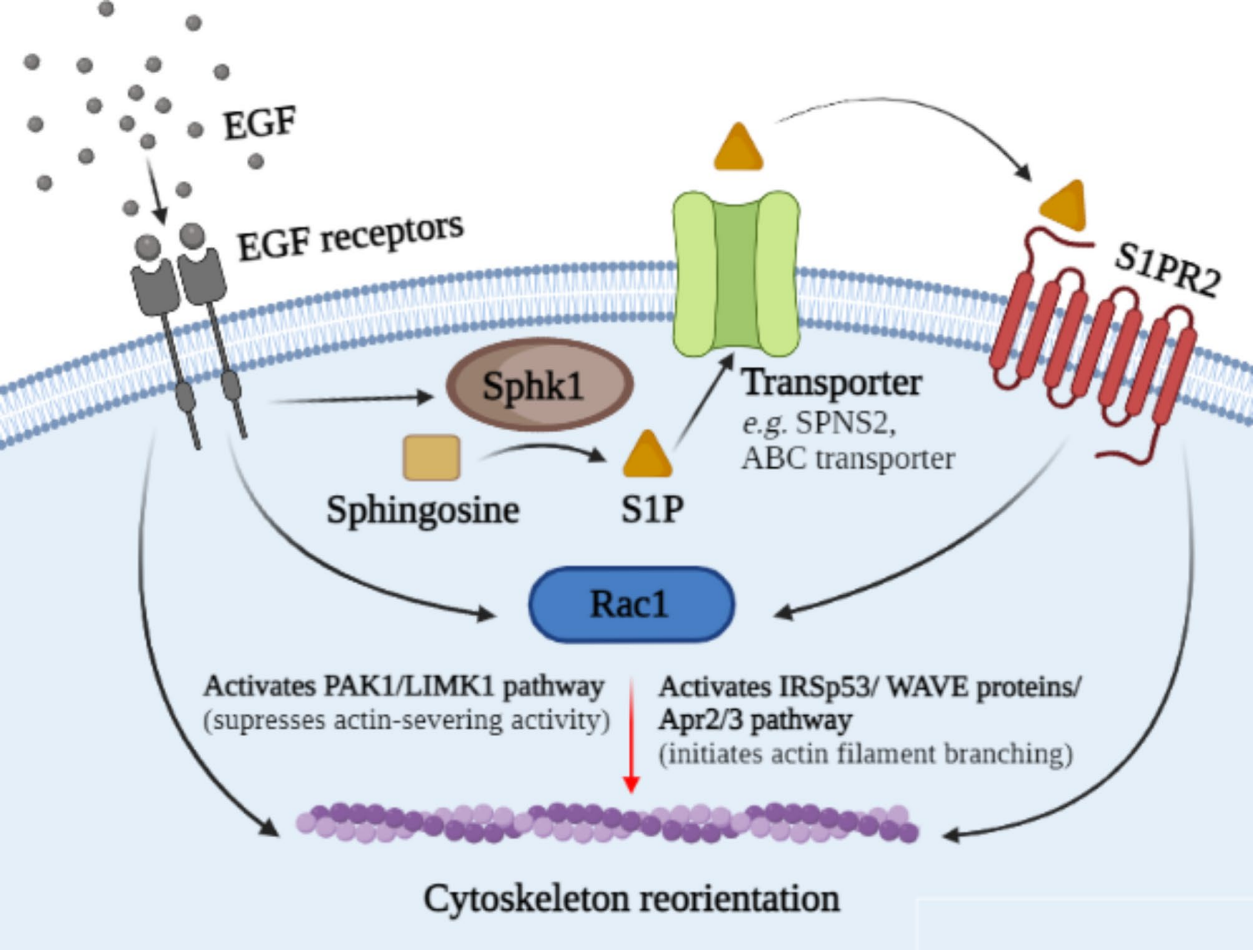
Proposed crosstalk between EGF and S1PR2 in H357, H400 and H413 cells. Sphk1 transcription is elevated following EGF treatment, resulting in the production of S1P. After being exported into the intercellular space, it binds to S1PR2, further generating a signal required for migration and invasion. EGF and S1PR2 co-ordinately regulates motility and Rac1 activity of H357, H400 and H413 cells. Rac1 controls cytoskeleton organisation via IRSp53/WAVE proteins/Apr2/3 pathway and PAK1/LIMK1 pathway, but this role (red arrow) has not yet been substantiated in the present study. (Diagram created using BioRender.com).

To evaluate the crosstalk between EGF/ EGF receptors and S1P/S1PR2 pathways, the metabolism of EGF and S1P was measured. This study also determined the production of TGF-β1 as this molecule is reported to have crosstalk with either EGF/EGFR and S1P/S1PR pathways [30, 42, 43]. TGF-β1 upregulated EGFR gene expression [42] and transactivated EGFR [30] as well as reportedly promoting S1P production by activating Sphk1 [43]. The result of the present study was consistent with the first model described above [25], as the activation of S1PR2 did not elevate the production of EGF or TGF-β1 in OSCC cultures (proposal crosstalk model summarised in Fig. 7). In this study, EGF induced S1P production only via Sphk1, and this agreed with a study [23] which reported that Sphk1 was the main enzyme that converts sphingosine into S1P. The S1P produced was then exported into the intercellular space to bind with the five S1PR subtypes which would then determine the function of the product. However, this was in contrast with a study of human cervical adenocarcinoma cells [44] which revealed that EGF/EGF receptors increased S1P production by activating Sphk2 within the endoplasmic reticulum or Golgi apparatus. Nevertheless, the present study indirectly determined the S1P production through the expression of mRNA, consequently it is possible that the alteration observed may not change the cellular S1P level significantly.

A limitation of this study is that the effect of S1PR2 was achieved only using chemical reagents. JTE013 is a selective S1PR2 antagonist and has been widely used at concentrations of up to 10 µM in a number of studies [6, 45–47], it has been reported that this reagent selectively inhibits the function of S1PR2 at concentrations lower or equal to 1 µM [48]. In a study on human breast cancer cells, JTE013 was also found to inhibit an S1PR4 antagonist [49]. Furthermore, for S1PR4, it has been reported to regulate migration and invasion pathways via epidermal growth factor receptor 2 (HER2) in breast cancer [49]. Taken together, there is a possibility that the inhibition of S1PR4 at high concentrations of JTE013 would cause the inhibition of cellular motility. In this case, the level and pattern of S1PRs expression of each cell type may therefore be another factor to consider. However, thus far no study has investigated which concentration of JTE013 would lose selectivity for S1PR2 in OSCC and determined the effect of S1PR4 on invasion in OSCC. Another limitation is that S1PR expression profile was determined using semiquantitative PCR, so it was unable to compare the level of expression between the different subtypes. Future studies should include this analysis to better clarify the role of S1P/S1PR signalling in OSCC.

In conclusion, S1PR2 and EGF in OSCC cultures appear linked in both function and metabolism. Migration and invasion of OSCC cells were inhibited following S1PR2 suppression, even after EGF stimulation. EGF itself could increase activation of S1PR2 through the upregulation of S1P production. This suggested that S1PR2 inhibitors could enhance currently available EGF receptor inhibitors as therapeutic targets in OSCC patients. Further work should also identify any potential off-target effects of S1PR2 inhibitors and this has also been proposed in other cell types [50, 51].

## Electronic supplementary material

Below is the link to the electronic supplementary material.

Supplementary Material 1
